# Enhancing survival predictions in lung cancer with cystic airspaces: a multimodal approach combining clinical and radiomic features

**DOI:** 10.3389/fonc.2025.1524212

**Published:** 2025-04-10

**Authors:** Liang Yin, Jing Wang, Pingyou Fu, Lu Xing, Yuan Liu, Zongchang Li, Jie Gan

**Affiliations:** ^1^ Medical Imaging, Shandong Provincial Third Hospital, Jinan, Shandong, China; ^2^ Radiology Department, Shandong Yellow River Hospital, Jinan, China

**Keywords:** lung cancer with cystic airspaces, survival, volume doubling time, radiomics, predictive model

## Abstract

**Objective:**

To enhance the prognostic assessment and management of lung cancer with cystic airspaces (LCCA) by integrating temporal clinical and phenotypic dimensions of tumor growth.

**Patients and methods:**

A retrospective analysis was conducted on LCCA patients treated at two hospitals. Clinical and imaging characteristics were analyzed using the independent samples t-test, Mann-Whitney U test, and χ^2^ test. Features with significant differences were further analyzed using multivariate Cox regression to identify independent prognostic factors. Radiomic features were extracted from CT images, and volume doubling time (VDT) was calculated from two follow-up scans. Separate predictive models were constructed based on radiomic features and VDT. A fusion model integrating radiomic features, VDT, and independent clinical prognostic factors was developed. Model performance was evaluated using receiver operating characteristic curve and the area under the curve, with DeLong’s test used for comparison.

**Results:**

A total of 193 patients were included, with an average survival time of 48.5 months. Significant differences were found between survivors and non-survivors in age, smoking status, chronic obstructive pulmonary disease, and tumor volume (*P* < 0.05). Multivariate Cox analysis identified smoking and chronic obstructive pulmonary disease as independent risk factors (*P* = 0.028 and *P* = 0.013). The VDT for survivors was 421 (298 582.5) days compared to 334.5 ± 106.1 days for non-survivors (*Z* = -3.330, *P* = 0.001). In the validation set, the area under the curve for the VDT model was 0.805, for the radiomic model 0.717, and for the fusion model 0.895, demonstrating the highest predictive performance (*P* < 0.05).

**Conclusion:**

Integrating VDT, radiomics, and clinical imaging features into a fusion model improves the accuracy of predicting the five-year survival rate for LCCA patients, enhancing personalized and precise cancer treatment.

## Introduction

Lung cancer remains one of the most lethal malignancies worldwide, with diverse histological subtypes and clinical presentations. Among these, lung cancer with cystic airspaces (LCCA), characterized by the presence of cystic airspaces within the lung parenchyma (1–3), presents unique diagnostic and therapeutic challenges. These cystic features can be visualized using imaging techniques such as computed tomography (CT) (4), but their atypical presentation often leads to misdiagnosis or delayed diagnosis (5, 6). Understanding the growth dynamics, clinical features, and imaging characteristics of LCCA is crucial for improving patient outcomes (7, 8).

Tumor volume doubling time (VDT) is a quantitative measure that reflects the growth rate of a tumor by calculating the time required for the tumor to double in size (9, 10). In the context of LCCA, VDT offers a potentially valuable metric for prognostic assessment (11). A longer VDT generally indicates a slower-growing tumor, which may correlate with a more favorable prognosis (12). However, several challenges hinder the clinical use of VDT, including variability in cystic measurements and the impact of factors such as fluid accumulation and air content on volume estimation (8). Additionally, the heterogeneity within LCCA, consisting of both solid and cystic components, complicates the precise determination of VDT. Radiomics, an emerging field that extracts high-dimensional data from medical images (13), holds promise for enhancing the prognostic evaluation of LCCA. By analyzing various quantitative features such as shape, texture, and intensity from CT or MRI images, radiomics can provide a comprehensive characterization of tumor heterogeneity and microenvironment (14, 15). These radiomic features have the potential to uncover patterns and biomarkers associated with tumor behavior and patient outcomes.

To overcome the limitations of using VDT or radiomics alone, this study integrates VDT, clinical data, imaging features, and radiomic analysis to improve the prognostic assessment of LCCA. This multifaceted approach aims to develop a robust and accurate prognostic model by combining temporal dynamics, clinical data, and phenotypic details.

The aim of this study is to advance the prognostic assessment and management of LCCA by uniting the temporal, clinical, and phenotypic dimensions of tumor growth, offering a novel pathway toward more precise and individualized cancer care.

## Materials and methods

### Study population

This retrospective, multi-center study was approved by the Institutional Review Board. Due to its retrospective nature, the requirement for individual informed consent was waived. The study was conducted at two medical centers: the Shandong Provincial Third Hospital and Shandong Yellow River Hospital.

We retrospectively analyzed the clinical and imaging data of patients diagnosed with cystic airspace-associated lung cancer based on surgical pathology from February 2015 to January 2020. The inclusion criteria were as follows: (1) LCCA confirmed by surgical pathology; (2) complete clinical, pathological, and imaging data; (3) at least two preoperative CT examinations with a minimum interval of six months, including the first and most recent preoperative CT scans; (4) no intrapulmonary metastasis or mediastinal lymph node metastasis; (5) a minimum postoperative follow-up period of five years (unless death occurred). The exclusion criteria were: (1) presence of another concurrent tumor or a history of malignancy; (2) history of tuberculosis or sarcoidosis; (3) receipt of preoperative anti-tumor treatments such as chemotherapy, radiotherapy, or immunotherapy; (4) postoperative complications; (5) poor image quality affecting analysis.

### CT acquisition

In this study, Institution I utilized two CT scanners for chest CT scans: a 128-slice scanner (Brilliance 64; Philips Healthcare) and a 256-slice scanner (Brilliance iCT; Philips Healthcare). Institution II conducted scans using two 64-slice scanners (Brilliance 64; Philips Healthcare and LightSpeed; GE Healthcare).

Patients were positioned supine with their hands raised above their heads, holding their breath at the end of deep inspiration. The scan range extended from the lung apices to the costophrenic angles. CT scan parameters included a tube voltage of 120 kVp, a tube current of 200–700 mAs, a slice thickness of 3 mm, and an inter-slice gap of 3 mm. Upon completion of the scans, routine reconstructions were performed for both lung window settings (window width: 1500 HU, window level: -500 HU) and mediastinal window settings (window width: 350 HU, window level: 40 HU), with a reconstruction matrix of 512 × 512.

### CT image analysis

The reconstructed images were uploaded to 3D Slicer 5.4.0 (https://www.slicer.org/) software for three-dimensional manual segmentation of the tumors. Two radiologists with 7 and 11 years of experience in thoracic diagnosis independently analyzed the images in a blinded manner. The inter-rater consistency of the region of interest (ROI) delineation was assessed using the intraclass correlation coefficient (ICC), with an ICC > 0.8 indicating good consistency. After segmentation, the software automatically calculated the nodule volumes.

The VDT was calculated based on the exponential growth model described by Schwartz, using the following equation (16): VDT = (T2 – T1) × log2/(logV2 – logV1), where T2 – T1 represents the time interval between two sequential CT scans, and V1 and V2 are the nodule volumes on the respective scans. To evaluate inter-reader variability in VDT measurement, the two radiologists independently repeated the VDT calculations on a random sample of 50 patients two weeks after completing the consensus assessment of all nodules.

### Radiomics analysis

To address the imbalance between the survivor and non-survivor groups, we applied the Synthetic Minority Over-sampling Technique (SMOTE) to the training set, generating synthetic samples for the minority class. Image normalization and radiomic feature extraction were performed in the pyradiomics package (https://github.com/AIM-Harvard/pyradiomics) within the Python environment. The radiomic features extracted from the ROI included first-order features, shape features (both 2D and 3D), gray-level co-occurrence matrix (GLCM) features, gray-level size zone matrix (GLSZM) features, gray-level run length matrix (GLRLM) features, gray-level dependence matrix (GLDM) features, and neighboring gray-tone difference matrix (NGTDM) features.

Due to the large number of radiomic features, dimensionality reduction was a critical step to prevent overfitting and enhance the model’s predictive efficiency. To achieve this, the least absolute shrinkage and selection operator (LASSO) algorithm was employed, leveraging its capability for both feature selection and regularization. A 10-fold cross-validation process was applied to determine the optimal lambda, minimizing the mean squared error. Radiomic features with non-zero coefficients at the selected lambda were retained, effectively reducing dimensionality while preserving relevant predictive information. Subsequently, a radiomic score (Radscore) was computed as a linear combination of the selected features, weighted by their respective coefficients. This Radscore served as a concise representation of tumor heterogeneity and was integrated into the predictive model.

### Clinical feature collection

Patient medical records were reviewed to collect information on lung cancer risk factors, including smoking, chronic obstructive pulmonary disease (COPD), occupational exposure, lung diseases, and family history of cancer. Morphological features were also recorded, including the classification of LCCA (17), the presence of spiculation, and preoperative tumor volume.

### Statistical analysis

Statistical analysis was conducted using SPSS 23.0 and R software (version 4.0.1, http://www.r-project.org). The *K*olmogorov-*S*mirnov test was used to assess the normality of quantitative data. Data following a normal distribution were expressed as mean ± standard deviation ( $\bar{x}$ ± s), while data not following a normal distribution were expressed as median (Q1, Q3). Comparisons between two groups were conducted using the independent samples t-test or the Mann-Whitney *U* test for quantitative data, and the χ² test for qualitative data.

Variables showing statistical significance in univariate analysis were included in the Cox regression analysis to identify independent prognostic factors for LCCA, using a stepwise forward selection method. Models based on tumor VDT, radiomics, and a combined model incorporating clinical risk factors were constructed.

Receiver Operating Characteristic (ROC) curves were plotted to evaluate the predictive performance of each model for LCCA survival, with the area under the curve (AUC) and the sensitivity and specificity at the optimal diagnostic threshold calculated. The DeLong test was used to compare AUCs. The combined model was visualized using a nomogram generated with the “rms” package, and its goodness-of-fit and calibration were assessed using calibration curves. Decision curve analysis (DCA) was employed to evaluate the clinical utility of the nomogram. The intraclass correlation coefficient (ICC) was used to assess the consistency of parameter evaluations by the two radiologists, with an ICC > 0.75 indicating good consistency. Statistical significance was set at a *P*-value of <0.05.

## Results

### Patient characteristics

This study included a total of 193 patients from two institutions, comprising 106 males and 87 females, with a mean age of 68.0 ± 7.5 years. The mean survival time was 48.5 months, ranging from 11 to 77 months. Of these patients, 153 survived for five years or longer, while 40 died within five years post-surgery. During the follow-up period, none of the 153 survivors developed tumor recurrence or metastasis. The 40 non-survivors died from tumor recurrence, metastasis, or other thoracic diseases. Based on five-year survival status, the patients were classified into two groups: survivors (n = 153) and non-survivors (n = 40).

### Univariate analysis and multivariate Cox regression analysis

Univariate analysis was conducted to identify clinical and imaging risk factors associated with the five-year survival rate. No significant differences were found between the survivor and non-survivor groups regarding gender, occupational exposure, ionizing radiation, genetic history, LCCA subtype, or the presence of spiculation (*P* > 0.05, **Table 1**). However, significant differences were observed for age, smoking, COPD, and tumor volume (*P* < 0.05, **Table 1**).

**Table 1:** Baseline clinical and imaging characteristics of survivors and non-survivors with LCCA.

Characteristics	Survivor (n=153)	Non-survivor (n=40)	Statistic Value	*P*-value
Age (years)	67.3 ± 7.6	70.6 ± 6.9	*t* = -2.459	0.015
Sex			*Χ^2^* = 0.606	0.436
Male	97 (63.4%)	28 (70%)		
Female	56 (36.6%)	12 (30%)		
Smoking			*Χ^2^* = 4.821	0.028
Smoker	59 (38.6%)	8 (20%)		
Non-smoker	94 (61.4%)	32 (80%)		
Ionizing Radiation				0.348
Exposed	6 (3.9%)	0 (0%)		
Unexposed	147 (96.1%)	40 (100%)		
Occupational Exposure				0.691
Yes	9 (5.9%)	1 (2.5%)		
No	144 (94.1%)	39 (97.5%)		
Genetic History				0.435
Yes	7 (4.6%)	3 (7.5%)		
No	146 (95.4%)	37 (92.5%)		
COPD			*Χ^2^* = 6.204	0.013
Yes	35 (22.9%)	17 (42.5%)		
No	118 (77.1%)	23 (57.5%)		
Spiculation			*Χ^2^* = 2.652	0.103
Yes	16 (10.5%)	8 (20%)		
No	137 (89.5%)	32 (80%)		
LCCA Subtypes			*Χ^2^* = 2.061	0.560
1	12 (7.8%)	2 (5%)		
2	63 (41.2%)	20 (50%)		
3	42 (27.5%)	12 (30%)		
4	36 (23.5%)	6 (15%)		
Tumor Volume (mm^3^)	1813.0 (1070.5, 2396.0)	2017.0 (1406.0, 2831.8)	*z* = -2.028	0.043

COPD, chronic obstructive pulmonary disease; LCCA, lung cancer with cystic airspaces.

Subsequently, multivariate Cox regression analysis was performed, incorporating the significant variables from the univariate analysis. The results confirmed that smoking and COPD were independent clinical risk factors (*P* < 0.05, **Table 2**).

**Table 2:** Results of cox regression analysis for clinical and imaging characteristics.

Variable	OR(95%CI)	*P*-value
Age	1.047 (0.999~1.096)	0.053
Smoking	2.769 (1.249~6.138)	0.012
COPD	2.172 (1.131~4.170)	0.020
Tumor Volume	1.000 (1.000~1.000)	0.213

COPD, chronic obstructive pulmonary disease; OR, odds ratio; CI, confidence interval;.

### VDT and radiomic models

**Figure 1:**
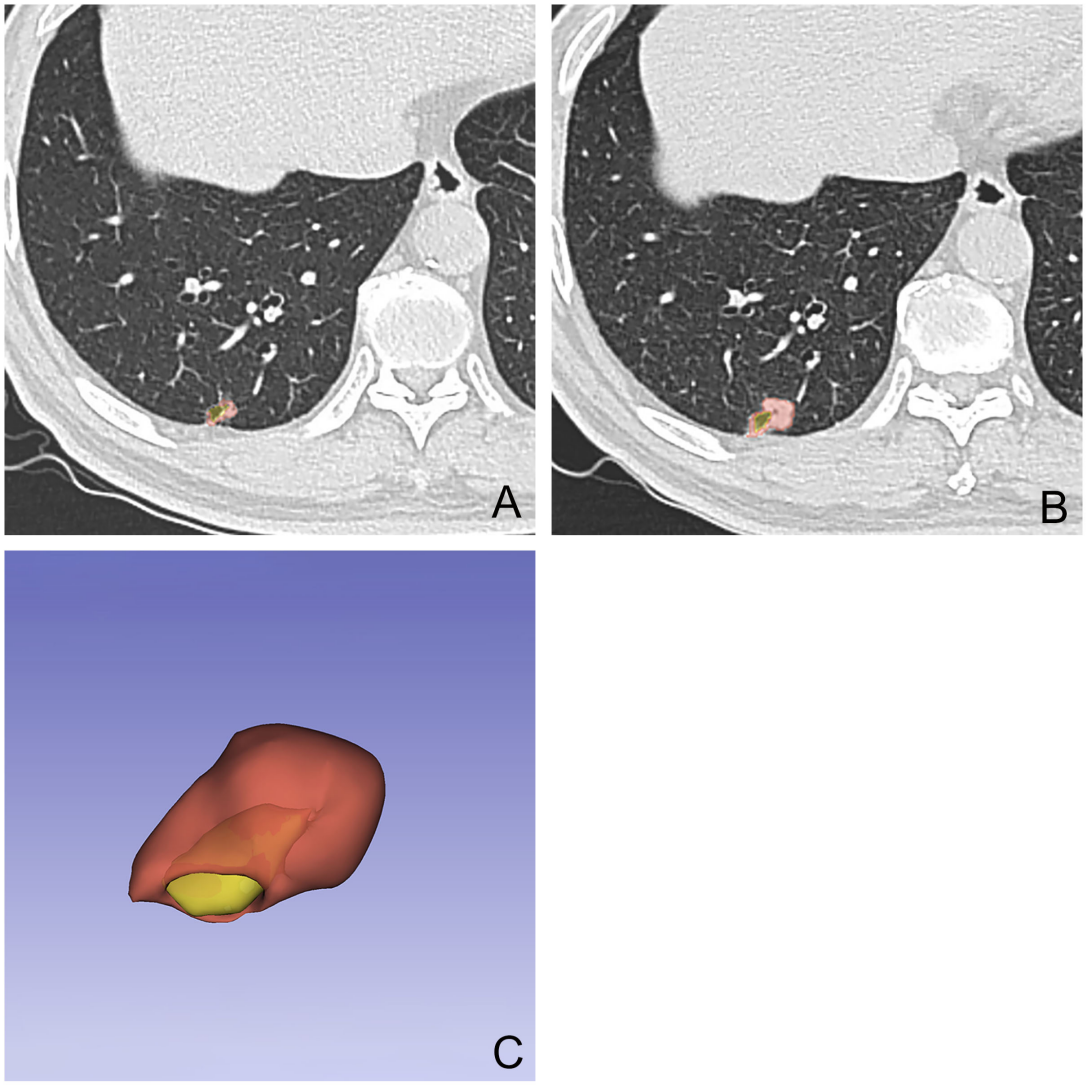
A 54-year-old male patient with a nodule with cystic airspaces identified during initial examination **(A)**. Follow-up CT at 12 months showed increased nodule size **(B)**. The final examination’s 3D tumor model displays the cystic area in yellow and the tumor in red **(C)**.

The inter-rater consistency for ROI delineation between the two radiologists was good (ICC > 0.80). Consequently, the ROIs delineated by the radiologist (L.Y.) with 11 years of diagnostic experience were used for VDT calculation and radiomic feature extraction (**Figures 1A–C**).

The VDT for the survivor group was 421 (298, 582.5) days, whereas for the non-survivor group, it was 334.5 ± 106.1 days, with the difference being statistically significant (*Z* = -3.330, *P* = 0.001).

Patients were randomly divided into a training set (n = 135) and a validation set (n = 58). There were no statistically significant differences in clinical and imaging characteristics between the training and validation sets (*P* > 0.05). From each ROI image, 851 radiomic features were extracted. ICC analysis retained 677 stable features, and the LASSO algorithm with 10-fold cross-validation was used to select 9 radiomic features to construct the radiomic signature. These included 1 shape feature, 1 texture feature, and 7 wavelet features. A Radscore was computed as a linear combination of the selected radiomic features and their weighted coefficients.

In both the training and validation sets, the Radscore of the survivor group was significantly higher than that of the non-survivor group (*P* < 0.001).

### Evaluation of predictive model performance

**Figure 2:**
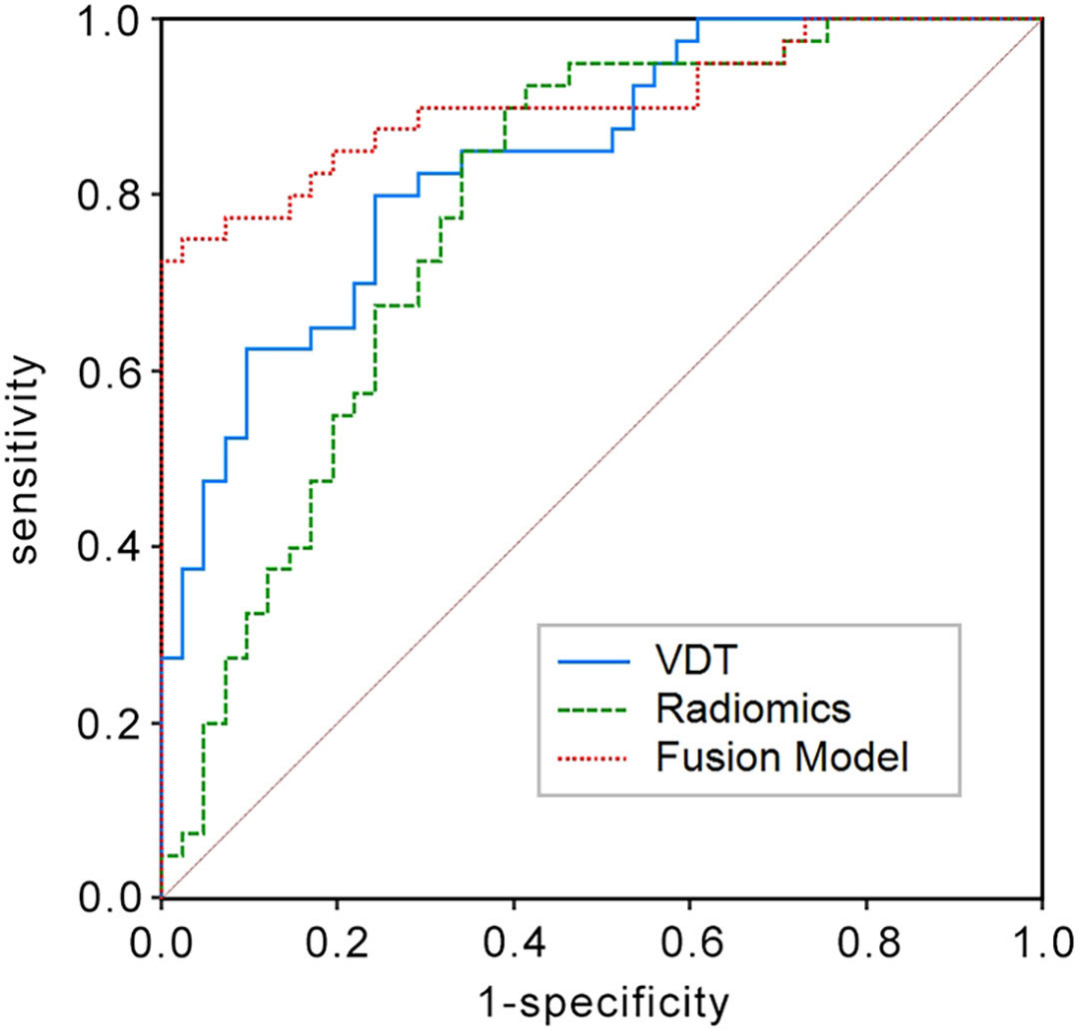
Receiver operating characteristic (ROC) curves in the training set for predicting the 5-year survival rate of LCCA using the Tumor Volume Doubling Time model, Radiomics model, and Fusion model. The Fusion model achieves the largest area under the curve (AUC = 0.905) among the three.

**Figure 3:**
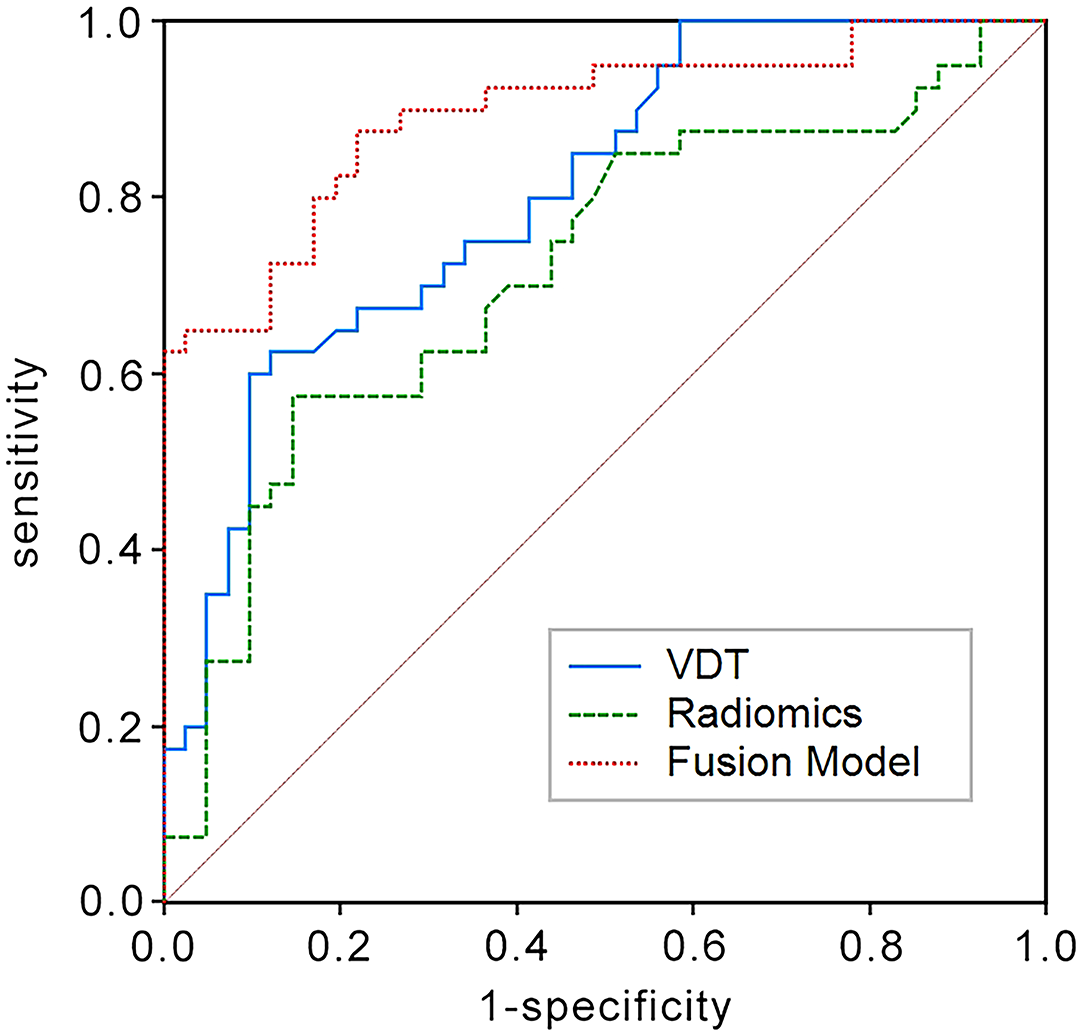
ROC curves in the validation set for predicting the 5-year survival rate of LCCA using the Tumor Volume Doubling Time model, Radiomics model, and Fusion model. The Fusion model again shows the highest AUC (AUC = 0.895) in comparison.

In the training set, the AUC for the VDT model in predicting the five-year survival rate of LCCA was 0.840, while the AUC for the radiomic model was 0.782. The fusion model, which integrated VDT radiomics, and clinical imaging features, achieved an AUC of 0.905 (**Figure 2**). In the validation set, the AUC for the VDT model was 0.805, and the AUC for the radiomic model was 0.717. The fusion model in the validation set achieved an AUC of 0.895 (**Figure 3**). In both the training and validation sets, the AUC of the fusion model was significantly higher than that of the VDT and radiomic models, with statistically significant differences (*P* < 0.05, **Table 3**).

**Table 3:** Efficacy of different models in predicting survival rates of LCCA in training and validation sets.

Group	AUC	95% CI	Sensitivity(%)	Specificity(%)	VS Fusion_Model	
					*Z*	P^*^
Training Set						
VDT	0.840	0.742 - 0.912	80.0	75.6	1.974	0.048
Radiomics	0.782	0.676 - 0.866	92.5	58.5	2.735	0.006
Fusion_Model	0.905	0.819 - 0.959	75.0	97.6		
Validation Set						
VDT	0.805	0.703 - 0.885	62.5	87.8	2.415	0.016
Radiomics	0.717	0.606 - 0.812	85.0	53.7	3.429	0.001
Fusion_Model	0.895	0.806 - 0.952	87.5	78.0		

VDT volume doubling time; AUC area under the curve; CI, confidence interval;.

*Delong test.

**Figure 4:**
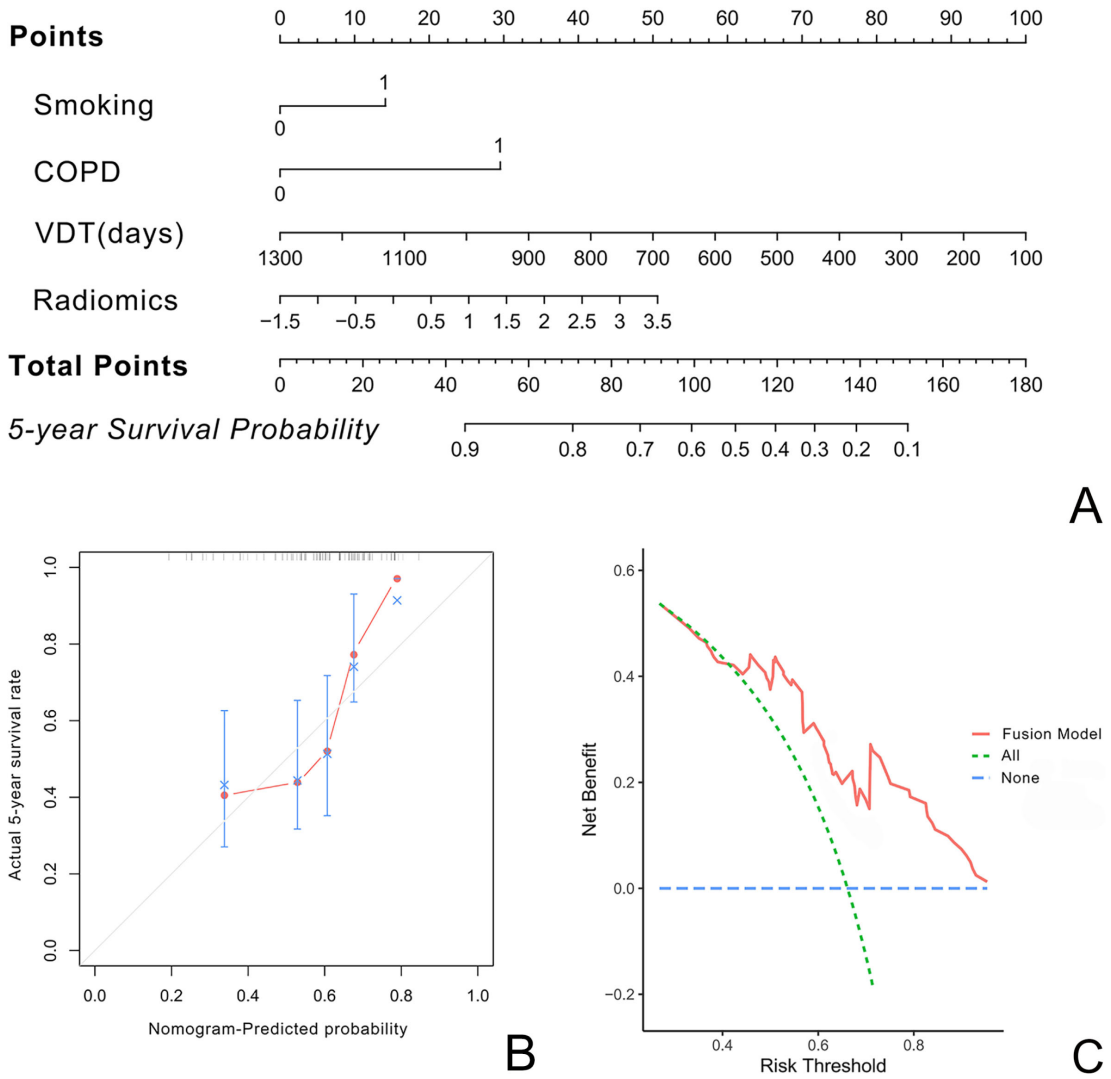
illustrate the nomogram, calibration curve, and decision curve for predicting the five-year survival rate of LCCA using the fusion model based on VDT, radiomics, and clinical imaging features. **(A)** presents the nomogram. **(B)** shows the calibration curve, indicating minimal deviation and demonstrating the high reliability of the fusion model. **(C)** displays the decision curve.

The nomogram constructed using the fusion model is shown in **Figure 4A**. Both the calibration curve (**Figure 4B**) and the clinical decision curve (**Figure 4C**) demonstrate the high reliability and consistency of the fusion model.

## Discussion

This study aims to establish a predictive model for the 5-year survival rate of patients with LCCA by integrating clinical and imaging-independent risk factors, tumor VDT, and radiomic features. The results indicate that among clinical and imaging characteristics, smoking and COPD are the only independent risk factors influencing the 5-year survival rate of LCCA patients. VDT and radiomic features play significant roles in survival prediction for LCCA patients, and the integrated model demonstrates higher predictive capability. Additionally, the constructed nomogram exhibits strong consistency in the calibration curve and DCA, making it a promising tool for clinical application.

Accurately assessing postoperative survival in LCCA patients remains challenging, despite advancements in the field (18, 19). Previous studies have primarily focused on clinical and imaging characteristics (4, 20). Snoeckx A et al. reported that the occurrence of LCCA is associated with smoking, with most cases reported among former and current smokers (21). COPD, characterized by chronic inflammation and airway damage (22), creates a favorable environment for the occurrence and progression of lung cancer. The presence of COPD is linked to poorer survival outcomes in lung cancer patients due to impaired lung function and increased susceptibility to complications (23). Lange C et al. confirmed that lung cancer patients with COPD have significantly lower survival rates than those without COPD (24). This study similarly identified smoking and COPD as independent risk factors for LCCA survival and incorporated them into the predictive model. Although Ma Z et al. found that different morphotypes of LCCA correlated with varying survival rates, with type 1 associated with excellent survival and type 2 with the worst prognosis (25), this study did not find statistically significant differences in survival in terms of morphotypes and thus did not include them in the final integrated predictive model.

VDT has proven beneficial in distinguishing high-risk from low-risk lung nodules in lung cancer screening (26, 27). Recent research highlights VDT’s importance in predicting lung cancer survival and treatment outcomes. Studies have validated VDT’s reliability as an effective tool and revealed its potential applications across different lung cancer types. Kakinuma R et al., in an eight-year observational study, confirmed that nodules with shorter VDT are more likely to be malignant, whereas longer VDT is associated with lower malignancy risk (28). Combining VDT with radiomic features, which provide detailed information on nodules’ shape, texture, and density, significantly improves the accuracy of predicting lung nodule malignancy (29). Consistent with previous findings, this study demonstrated that combining VDT with radiomics and clinical imaging features results in a more comprehensive and reliable predictive tool, paving the way for personalized treatment approaches.

The study found that the integrated model, which combines VDT, radiomics, and clinical imaging features, outperforms individual VDT and radiomic models in predicting the 5-year survival rate of LCCA patients. This comprehensive approach leverages the strengths of multiple data types to enhance prognostic accuracy, with recent literature increasingly supporting this method (30, 31). Compared to standalone VDT and radiomic models, the integrated model exhibits higher AUC values. Limkin EJ et al. showed that integrating clinical, imaging, and molecular data significantly improves the accuracy of cancer prognosis models (32). Their findings align with this study’s results, indicating that models incorporating diverse data types perform better than those based on a single data source. The predictive model developed in this study exhibits high accuracy, sensitivity, and specificity, with minimal bias observed in the calibration curves, underscoring the model’s reliability. This approach enhances the accuracy and reliability of survival predictions for LCCA patients, supporting more informed clinical decisions and personalized treatment strategies.

However, the study has certain limitations. Firstly, the sample size may be relatively small, particularly for rare types of LCCA, potentially leading to overfitting. Second, although this is a dual-center study, external validation with larger, multi-center cohorts is necessary and will be prioritized in future research. Thirdly, as a retrospective study, it may face selection bias. Lastly, the manual tumor delineation, although consistent among physicians, is time-consuming and may benefit from automated or semi-automated methods to enhance efficiency and stability (33).

In conclusion, integrating VDT, radiomics, and clinical imaging features into a fusion model provides a more accurate and reliable method for predicting the 5-year survival rate of LCCA patients, enhancing the potential for personalized and precision cancer treatment.

## Data Availability

The raw data supporting the conclusions of this article will be made available by the authors, without undue reservation.
